# Self-Reported Halitosis in relation to Oral Hygiene Practices, Oral Health Status, General Health Problems, and Multifactorial Characteristics among Workers in Ilala and Temeke Municipals, Tanzania

**DOI:** 10.1155/2017/8682010

**Published:** 2017-02-09

**Authors:** C. M. Kayombo, E. G. Mumghamba

**Affiliations:** Department of Restorative Dentistry, School of Dentistry, Muhimbili University of Health and Allied Sciences (MUHAS), P.O. Box 65014, Dar-es-Salaam, Tanzania

## Abstract

*Aim.* To assess self-reported halitosis, oral hygiene practices, oral health conditions, general health problems, sociodemographic factors, and behavioural and psychological characteristics among workers in Ilala and Temeke municipals.* Materials and Methods.* This was a cross-sectional descriptive study. Four hundred workers were recruited using a self-administered structured questionnaire.* Results.* Self-reported tooth brushing practice was 100%, tongue cleaning 58.5%, dental flossing 4.3%, gum bleeding on tooth brushing 79.3%, presence of hard deposits on teeth 32%, mobile teeth 15.3%, and self-reported halitosis (SRH) 48.5%. Tea users were 95%, coffee users 75.8%, smokers 21%, and alcohol consumers 47%. The SRH was significantly associated with bleeding gums, hard deposits, and mobile and malaligned teeth. Tongue cleaning and regular change of toothbrush were associated with low prevalence of SRH (*P* < 0.001). Higher occurrence of SRH was significantly related to low education and smoking.* Conclusion.* Self-reported halitosis was prevalent among workers and was significantly associated with bleeding gums, hard dental deposits, mobile teeth, and smoking. All participants brushed their teeth and cleaned the tongue regularly but use of dental floss was extremely low. Oral health education and health promotion are recommended.

## 1. Introduction

Oral hygiene is the practice of keeping the mouth and teeth clean to prevent dental problems, most commonly, dental cavities, gingivitis, periodontal (gum) diseases, and bad breath [1].

Halitosis, oral malodour, or bad mouth breath is a universal medicosocial problem in all communities and refers to the unpleasant odour that originates from the mouth or elsewhere [2]. Most of the times these three “terms” had been used interchangeably in literature and likewise they have been adopted in the current report. The condition is multifactorial in aetiology and may involve both oral and nonoral conditions.

Halitosis is a nuisance problem for many people around the world. It affects social interactions of people in daily life by causing personal discomfort and emotional stress. Previous studies have reported that about 30% to 50% of the population has experienced the problem of halitosis [3–5].

Halitosis has been classified into three main categories: genuine, pseudo-halitosis, and halitophobia halitosis [6]. In genuine halitosis, the malodour intensity is beyond the socially acceptable level. If halitosis is not perceived by others but the patient persistently complains of its existence, it is diagnosed as pseudo-halitosis. If after successful treatment of genuine halitosis or pseudo-halitosis the patient still complains of halitosis, the diagnosis is referred to halitophobia [6]. A term psychosomatic breath malodour (halitophobia, pseudo-halitosis) is used when breath malodour does not exist in actual fact but the patient imagines and believes that he or she has breath malodour. Halitophobia cannot be objectively determined and is mostly associated with suicidal attempts [6, 7]. Genuine breath malodour from the oral cavity contains volatile sulphur compounds (VSCs) particularly hydrogen sulphide, methyl mercaptan, dimethyl-sulphide, and organic acids [6, 8].

The causes for breath malodour (BM) are multifactorial in that it may arise from dental plaque, bacterial products from deep periodontal pocket, tongue, tonsils and pharynx, and rarely gastrointestinal tract [8]. Breath malodour is also associated with gingival bleeding on tooth brushing [9] and higher number of bleeding sites on probing [10]. Oral prosthetics such as acrylic dentures, especially when retained in the mouth at night or are poorly and irregularly cleaned, can also produce a typical smell associated with candidiasis [11].

Nonoral causes for genuine breath malodour include medical problems such as renal failure, cirrhosis of the liver, and diabetes mellitus [3, 11, 12]. Although breath malodour can originate from oral and nonoral sites, about 85% are generally related to an oral cause [3, 11].

Major methods of analysing BM include organoleptic measurement (judges for BM), gas chromatography, and sulphide monitors [6, 13]. In addition to these methods, clinical application of a questionnaire for diagnosis and treatment of breath malodour has been developed for use [14].

Poor oral hygiene not only is closely linked to various oral health problems but also has a significant effect on oral malodour. Mechanical tooth cleaning, such as tooth brushing or interdental flossing, is an essential daily oral hygiene practice, but many articles have revealed that tooth brushing alone will not significantly reduce oral malodour [15]. On the other hand, mouth rinsing and tongue cleaning can reduce VSCs levels [16].

The information on oral malodour in African countries including East Africa particularly in Tanzania general population is scarce. The prevalence of self-reported halitosis was 72% among adolescents in Temeke district [17], fourteen percent among young women at maternity block in Muhimbili National Hospital [9], and 44% in Muhimbili outpatient dental clinic [18]. Most of the studied outpatients (66%) at Muhimbili Dental Clinic were of the opinion that oral malodour was a problem in their society, and the majority of these respondents (64.5%) were residents in Kinondoni district [18].

The aim of the current study was to determine the self-reported halitosis, oral hygiene practices, oral health conditions, general health problems, and sociodemographic, behavioural, and psychological characteristics among workers in Ilala and Temeke municipals.

## 2. Materials and Methods

### 2.1. Study Design, Place of Study, and Participants

This was a cross-sectional descriptive study. The study was conducted in Ilala and Temeke municipals, Dar-es-Salaam, Tanzania, which were readily accessible and thus conveniently selected whereby rural areas were not part of this study. All workers found in business sites in particular the factories, garages, shops, offices, and schools on the day and time of data collection were eligible to participate. The workers were either self-employed or employed for pay. Only those who consented were included in the study.

### 2.2. Data Collection Tool and Data Management

A self-administered structured questionnaire that was formulated first in English and then translated into “Swahili,” the local language of the study participants, was distributed physically by the researcher (CK) to all workers readily available on the study sites. No sampling was done. Recruitment of study participants was done by registering consecutively every consenting person at different work stations until the sample size was attained. The questionnaire had open- and closed-ended questions composed of inquiry items on sociodemographic characteristics, dental caries experience, oral hygiene practices, self-reported halitosis (SRH), periodontal diseases, general systemic health problems, and behavioural factors in particular cigarette smoking and alcohol consumption (see the questionnaire in the in Supplementary Material available online at https://doi.org/10.1155/2017/8682010).

Data was entered into a computer and analysed using Statistical Package for Social Sciences (SSPS) version 20.0. Frequency and cross-tabulation tables were generated. Data transformation was undertaken and it included dichotomization of some variables that had more than two options for example, age (less than 30 years versus 30 years and above or at age group of 40 years and above compared to those that were less than 40 years of age), level of education, and type of education (primary education and lower versus secondary and higher education). The type of occupation of individuals was transformed, dichotomized, and recoded as “indoor” versus “outdoor” workers. Chi-Square test or Fisher's Exact Test was used to detect associations given with the Chi-squared (*χ*^2^) value truncated at two decimal points. In all the analyses, the statistical significance level was set at “*P* < 0.05.” For the logistic regression analyses, a total of thirty-seven multifactorial characteristics were included in “back-ward model” to determine their impact on the self-reported halitosis whether they had statistically significant contribution or not.

### 2.3. Ethical Considerations

This work was an elective study which was part of the requirement for the doctor of dental surgery (DDS) undergraduate training at the Muhimbili University of Health and Allied Sciences (MUHAS). Ethical clearance was granted by the Research and Publication Committee of the School of Dentistry as empowered by MUHAS Ethical Committee.

## 3. Results

### 3.1. Study Participants

A total of 400 workers were recruited as study participants and aged 17–59 years (mean = 35.7 ± 9.0) years with the median of 34.5 years (Table 1). The level of education of the participants was primary (33%), secondary (38%), and college (24.3%) education. Only 4.8% had no formal education.

### 3.2. Self-Reported Halitosis in relation to Oral Hygiene Practices, Oral Health Status, and General Health Problems

All participants claimed to practice regular tooth brushing but frequency of tooth brushing varies among individuals. Tooth brushing once per day was 43% while twice or more per day was 57%. The prevalence of SRH was 48.5% and experienced in different times of the day and occasions (Table 2). About one-fifth (21.7%) of the participants had different general medical conditions that include cardiovascular diseases (CVD) in particular heart problems and hypertension (8.5%), endocrine disorders especially diabetes (4.3%), and other systemic conditions (Table 3). The participants that had CVD that includes hypertension exhibited higher SRH than those that had other systemic conditions (11.3% versus 5.8%, *χ*^2^ = 3.91, *P* = 0.048; table not shown). Among the participants who brushed their teeth twice per day or more had lower proportion (36.0%) of SRH compared to those (65.1%) who had brushed their teeth just once per day (*χ*^2^ = 33.35, *P* < 0.001). A total of 399 (99.75%) used plastic toothbrush (the participants who used plastic toothbrush alone were 87%, both plastic as well as chewing stick 12.75%), and chewing stick alone only 1 person (0.25%). There was no significant difference in the occurrence of SRH between the participants who used both plastic toothbrush and chewing stick compared to those who used plastic toothbrush alone.

The SRH in relation to oral hygiene practices, oral health status, and general health/medical problems is shown in Table 4. The SRH was higher among those who used the toothbrush for four months or more before changing it compared to those that used it for three months or less (*χ*^2^ = 13.93, *P* < 0.001). All study participants used at least one type of a dentifrice among the nine listed as available in the shops which included Aha, Aloe vera, Chemi-dent, Chinese-brands, Close-up, Colgate, Sensodyne, Traditional herbs, and White-dent. For ethical reasons these dentifrices were randomly coded as toothpaste (TP) type A-I. Toothpaste type B and type E users had lower proportion of participants that had SRH as compared to nonusers *χ*^2^ = 4.13, *P* = 0.042 and *χ*^2^ = 5.42, *P* = 0.02, respectively. Interestingly, some participants used charcoal (8.8%), ashes (1.5%), and/or sand (0.3%) to clean their teeth. Among those who used charcoal, a higher proportion of participants complained of SRH compared to those who did not use charcoal (*χ*^2^ = 4.55, *P* = 0.033).

The participants who brushed their teeth before going to bed had lower proportion of SRH than those who did not do so (*χ*^2^ = 30.83, *P* < 0.001). For those that had the habit of not cleaning the tongue, they had more complains of SRH compared to those who cleaned their tongues (*χ*^2^ = 35.85, *P* < 0.001). The participants that had food stuck between teeth exhibited higher SRH than those without stuck food (*χ*^2^ = 6.16, *P* = 0.013). Dental floss users to clean the spaces between the teeth were very few (2.5%). Toothpick users (19.0%) had higher SRH than nonusers (*χ*^2^ = 6.32, *P* = 0.012), and the same trend was observed between participants that had teeth malalignment due to increasing space between teeth compared to those without (*χ*^2^ = 9.28, *P* = 0.002, Table 4). Gum bleeding on tooth brushing resulted to more complaints on SRH compared to those who had no bleeding (*χ*^2^ = 39.97, *P* < 0.001). Hypertensive individuals had higher SRH compared to those that had other systemic conditions (*χ*^2^ = 3.94, *P* = 0.047, Table 4).

Sociodemographic factors together with behavioural and psychological characteristics in relation to SRH are shown in Table 5. The participants that had primary education or less exhibited significantly higher proportion of SRH than those that had secondary education or higher (*χ*^2^ = 14.99, *P* < 0.001). Similar trend was evident among participants at age group of 40 years and above compared to those that were less than 40 years (*χ*^2^ = 4.07, *P* < 0.044). There were no significant differences in the prevalence of SRH between males and females and likewise between the married and singles.

A total of 157 out of 400 (39.2%) were outdoors workers (casual labourers, drivers, security guards, garage people, petty business persons, and porters) and 60.8% were indoor workers (managers, administrators, accountants, secretaries, receptionists, office attendants, teachers, nurses, industrial engineers, and laboratory technicians). There were more outdoors workers that had SRH compared to indoor workers (*χ*^2^ = 13.38, *P* < 0.001). Likewise, SRH was higher among smokers compared to nonsmokers (*χ*^2^ = 5.780, *P* = 0.016). The participants that had opportunity to come closer to someone that had halitosis influenced them to an extent that most of them perceived SRH than those that had not met anyone with such situation (*χ*^2^ = 16.49, *P* < 0.001).

Among the 194 participants that had SRH problem, more than two-fifth (41.3%) discovered it through gestures from people near them when talking and 23.8% were informed by their spouses (30.8) and relatives (23.8%, table not shown). Furthermore, among those affected by SRH, 77.3% hesitated to talk to other people, 41.2% tried to stay away from other people, but also 54.1% thought that other people were shunning away from them (Table not shown). Almost all (99.5%) of the participants that had SRH were bothered to an extent that it had affected them at work place (*χ*^2^ = 241.9, *P* < 0.001) as well as at home (*χ*^2^ = 273.1, *P* < 0.001; Table 5). Use of chewing gum was significantly higher among the SRH group compared to those without the problem (*χ*^2^ = 7.16, *P* < 0.01, Table 5). Self-treatment for SRH was attempted by 46.9% of the participants whereby 24.2% used chewing gums and mouth-washes (31.4%), and 16.0% tried to seek doctor's advice. For the participants that had SRH, when asked, “are you willing to be treated for SRH problem”, 95.9% responded positively. The difference between those willing compared to those not willing to be treated was highly significant (*χ*^2^ = 318.82, *P* < 0.001).

### 3.3. Self-Reported Halitosis in relation to Previous Tooth Extraction

The SRH was higher among participants that had had tooth extraction than in those who had no extraction (*χ*^2^ = 4.72, *P* = 0.03, table not shown). Likewise, the same trend was seen among those that had tooth extraction due to dental caries compared to those that had extraction of teeth without tooth decay (*χ*^2^ = 8.77, *P* = 0.003).

### 3.4. Knowledge on the Origin of Halitosis

Those participants that experienced SRH thought that the problem was due to bleeding gums on tooth brushing (70.1%), not brushing the teeth well (67.0%), holes on teeth (decayed teeth) (41.8%), and dry mouth (20.1%).

### 3.5. Logistic Regression Analyses

The binary logistic regression analyses of the multifactorial characteristics related to self-reported halitosis among the participants are shown in Table 6. Multifactorial characteristics (dichotomized) that were considered and entered in the binary logistic regression back-ward model were those dealing with halitosis in the bivariate analyses above (Tables 4 and 5) in relation to oral hygiene practices, oral health status, and sociodemographic characteristics as well as behavioural and psychological factors. The logistic regression model process went through many steps whereby at each step some of the variables were removed from the model and the final step with few variables that were maintained in the model with Odds ratio (OR), 95% confidence interval (CI), and Probability (*P*) value in three decimal points (Table 6). In this table, it can be seen that a closer interaction with someone that had halitosis was significantly associated with more than four times likelihood to declare SRH (OR: 4.19, CI 1.61–9.69, and *P* = 0.003) compared to an individual that had never met anyone with a problem of halitosis. Also those participants that hated the type of job they were doing had more than two times likelihood to declare SRH (OR: 2.31 (CI 1.02–5.21), *P* = 0.044) than the ones, for example, who had interesting jobs. Those who were married were less likely to report SRH than those who were single (OR: 0.54, (CI 0.31–0.94), and *P* = 0.031). Also participants at the level of primary education and lower were less likely to report SRH than those that had higher level of education from secondary school and above. The participants that had poor oral hygiene in particular those not brushing their teeth before bed as well as those not cleaning the tongue were less likely to assert SRH compared to their counterparts, OR: 0.36, (CI 0.21–0.60), and *P* < 0.001 and OR: 0.51, (CI 0.30–0.88), and *P* = 0.016, respectively. Likewise, those that had oral health problems like bleeding gums on tooth brushing or had dental hard deposits were less likely to claim having SRM compared to those without those conditions, OR: 0.21, (CI 0.10–0.41), and *P* < 0.001 or OR: 0.32, (CI 0.18–0.57), and *P* < 0.001, respectively.

## 4. Discussion

Halitosis is a common problem among general population and evidences reveal that it forms about 85% of all complaints when considering extraoral origins and psychological types [19]. In many studies, including ours, the assessment of halitosis relies on the subject's self-perception. Many professionals do not consider this method to be reliable because it is subjective, and obviously, the method is not standardized among participants. Although the method presents several problems and may be objectionable to the dentist, it is the one that most closely resembles daily situations in which halitosis is detected [20]. The prevalence of halitosis, according to the studies published, is between 2% and 44% and this disparity is justified by the subjectivity of the diagnostic criteria, assessment methods, and sampling techniques [20].

In the present study the overall prevalence of halitosis was approaching fifty percent; this is almost similar to the results reported in Qassim, Saudi Arabia [21], is also lower in comparison to study done in Kinondoni [22], but at the same time is higher than the findings reported in other populations especially Brazil [23] and USA [20]. As far as oral hygiene practices are concerned all participants practiced tooth brushing and this could suggest that people had knowledge on how to take care of their oral cavities. However, this cannot be taken for granted that they were practicing tooth brushing correctly, the subject matter that was beyond the scope of this study. It is known that adequate oral hygiene measures may reduce or treat people suffering from halitosis or protect them from it [24]. Consistent with the previous studies [25, 26], lower frequency of tooth brushing in the current study was related to higher occurrence of halitosis. Longer use of a tooth brush more than three months was associated with higher occurrence of halitosis and this could be explained by the fact that cleaning effectiveness of the bristle brushes diminishes with time of use and that changing the toothbrush after every use leads to decrease of microbes responsible for plaque formation [27]. The toothbrush has a significant role to reintroduce microorganisms into the oral cavity [27, 28]. Since it is not feasible to change the toothbrush every day, it is recommended as a sound practice to change the toothbrush at least after every three months [29].

The tongue is said to be the most common source for halitosis within the oral cavity [20]. In the current study the halitosis was significantly higher among those who did not clean the tongue compared to those who did and this concurs with previous reports [23, 30]. The possible explanation is that the uncleaned tongue usually harbours periodontal bacteria such as* Prevotella intermedius*,* Porphyromonas gingivalis*, and* Fusobacterium* species that are responsible for producing volatile sulphur compounds (VSC) that account for halitosis [30].

In the current study, participants who brushed their teeth before going to bed had significantly lower prevalence of halitosis than those who did not brush their teeth before going to bed. It may be considered that a major reduction in microbial plaque before going to bed will result into a lower total number of the microbes responsible for halitosis and its intensity bearing in mind that one microbe in 24 hours multiplies after every 3 hours and ends up in totaling into 254 [31]. A multitude of microorganisms of different species are present in the oral biofilms as it is estimated that 1 mm^3^ of dental plaque weighing about 1 mg, more than 10^8^ bacteria, may be counted [31]. There are reports showing that the dentifrice containing triclosan and copolymer in a sodium fluoride/silica base reduces the number of VSC-producing bacteria [32] and that the concentration of triclosan in plaque biofilm inhibits the growth of bacteria and therefore retards the return of halitosis [33]. Therefore, the use of certain toothpastes, especially fluoridated toothpaste, should be recommended for not only dental caries prevention but also managing halitosis [32]. Use of some dentifrices in the current study was associated with lower prevalence of halitosis and this is in a way in line with what has been reported elsewhere [33, 34]. Periodontal diseases and dental caries are potential factors contributing to halitosis [35].

Gum bleeding on tooth brushing and hypertension were associated with higher prevalence of halitosis in the study population and putrefaction of the blood in the gingival sulcus or periodontal pocket might be the possible explanation for the observation [11]. Furthermore, high blood pressure might contribute to pumping of more blood to the gingival sulcus or periodontal pocket area and thus they are more readily available for putrefaction. Self-reported presence of decayed teeth in the present study was associated with high prevalence of halitosis and corroborates with Tin-Oo and coworkers study in Malaysia [36] but is contrary to Eldarrat et al. findings in Libya [35]. Essentially any oral site in which microbial accumulation and putrefaction can occur may be an origin for halitosis. In addition to the most common intraoral sites for halitosis production (the tongue, interdental, and subgingival areas), other foci may include faulty dental restoration sites, sites of food impaction, and abscesses [37]. In the current study the halitosis was significantly higher among those who had food impaction between teeth compared to those who had none and concurs with previous study in Nigeria [38].

Similar to the SRH study in Kuwait [25] and Turkey [39] participants that had lower level of education in the current study in Temeke and Ilala municipality had higher prevalence of halitosis and might be explained by the low level of understanding in such groups. Self-reported halitosis in those over 30 years of age was higher than those under 30 although the difference did not reach a statistically significant level; in a way it shows an inclination to previous studies which reported significantly higher SRH in those over 30 years of age than those less than 30 years [21, 40, 41]. In current study, halitosis was not associated with sex and similar findings had been reported in Turkey [39], Thailand [40], and Saudi [42]; however, opposite findings show that males were affected more than females in Brazil [23], Poland [26], and Saudi Arabia [43] and vice versa that females were more affected than males in Italy [44].

Halitosis can have a distressing effect that may become a social handicap and the affected person may avoid socializing [35]. In this study, almost all subjects that had halitosis admitted that it interfered with their social life and significantly was noted at home, in the evening and at night and these parameters suggest personal discomfort and social embarrassment [22]. Patients with SRH chose to share this problem with friends, relatives, and others more frequently than with health professionals [40]. In the present study, self-treatment was found to be higher than the one reported in Saudi Arabia [43]. About a quarter of the participants in the current study used chewing gums and mouth-washes for self-treatment of halitosis. Chewing gum has been reported to have the ability to significantly increase salivary flow rate, raise the plaque-pH levels, and improve halitosis [32]. Most of the participants that had halitosis in the current study were using chewing gum and similar observation has been reported by Fadhil and Mugonzibwa [18].

Among the participants that had SRH, an interesting observation was that some (a small proportion) were not willing to be treated for their problem. The reason for this is not known but it can be speculated that dental fear might be the contributing factor as is a common phenomenon in the world as more than 25% of patients avoid visits and treatments, and approximately 10% reach phobic levels of anxiety [45]. Most of those with halitosis were willing to be treated. This is a good sign that most of those affected want to eliminate the embarrassing problem but, on the other hand, needs to ring bell to the practitioners to be well equipped to manage adequately both the genuine and even the psychological halitosis [46].

Smoking has been defined as an extrinsic cause of oral halitosis [47]. In the current study smokers showed significant association with halitosis similar to other previous reports [23, 44, 48]. Smoking has been implicated to decrease olfactory sensitivity [43] and this might have a negative impact that it may limit the identification of self-reported halitosis.

Alcohol ingestion may result in transient halitosis because some substances can cause xerostomia and alcoholic beverages are known to produce volatile compounds, acetaldehyde, and other odorous by-products by oxidation of alcohol in the mouth and liver [49]. However, similar to previous studies [22, 40], participants who consumed alcohol showed no association with self-reported halitosis. Contrary to our findings, it has recently been reported that alcohol intake could be considered as an important predictor for halitosis [50].

Interpretation of the findings of this study needs consideration in some few important limiting factors based on the fact that, first, the data was based on self-reported information that could not be verified clinically. Second, the study was voluntary, so some of the workers that were shy and afraid of embarrassment might have avoided themselves from participating in the study after knowing that halitosis was the topic of interest leading to failure to capture every one that was present during data collection. Third, for the participants that reported not to have halitosis, it was not certain whether they were free from the problem due to the fact that it was an embarrassment to tell someone that she or he has such a problem.

## 5. Conclusion

The self-reported halitosis in the studied population was substantial and the affected ones demonstrated willingness to be treated. Older age, not cleaning the tongue, food impaction between teeth, low education level, smoking, high blood pressure, change in alignment of teeth, and bleeding during tooth brushing were the factors significantly associated with the self-reported halitosis. All participants reported daily tooth brushing with dentifrice but practiced limited interdental flossing. The public is probably not fully aware of the potential causes of halitosis and its management.

## 6. Recommendation

Further research on most appropriate and feasible methods to diagnose halitosis and its management including prevention is recommended.

## Supplementary Material

The file contain the questionnaire used in the study, the questionnaire has open ended and closed ended questions. The questionnaire was formulated in English then translated into Swahili which was the one used for all the participants.

## Figures and Tables

**Table 1:** Distribution of the study participants by age groups and sex.

Age group	Males		Females		Total	
	*n*	%	*n*	%	*n*	%
17–29	59	26.6	59	33.1	118	29.5
30–39	82	36.9	69	38.8	151	37.8
40–49	59	26.6	37	20.8	96	24.0
50–59	22	9.9	13	7.3	35	8.8

Total	222	(100)	178	(100)	400	(100)

**Table 2:** Self-reported halitosis in relation to time in a day when it is experienced more.

Time of a day	Problem with halitosis				Chi-square test	
	Yes		No			
	*n*	%	*n*	%	*χ* ^2^	*P* value
After waking up	145	74.7	14	6.8	192.6	0.000
When talking to others	92	47.4	7	3.4	104.0	0.000
When fasting	90	46.4	3	1.5	113.1	0.000
During work	49	25.3	2	1.0	53.0	0.000
When thirst	40	20.6	5	2.4	33.1	0.000
In the afternoon	46	23.7	2	1.0	48.9	0.000
In the evening	43	22.2	1	0.5	48.0	0.000
At night	40	20.6	1	0.5	44.0	0.000
When tired	52	26.8	2	1.0	56.8	0.000
The whole day	88	45.4	4	1.9	106.4	0.000

**Table 3:** General medical conditions reported by the study participants.

General medical problems of the study participants	*n*	%
Cardiovascular diseases: hypertension and heart problems	34	8.5
Endocrine disorders: diabetes, thyroid conditions	17	4.3
Respiratory tract (RT) system: tuberculosis (TB), RT infections, asthma	8	2.0
Central nervous system: headache, psychosomatic, and eye problems	11	2.8
Gastrointestinal tract problems	6	1.5
Genitourinary tract problems	2	.5
Musculoskeletal system: backache and joint problems, skin	9	2.3
No medical problem	313	78.3

Total	400	100.0

**Table 4:** Bivariate analysis: self-reported halitosis in relation to oral hygiene practices, oral health status, and general health problems.

^#^Multifactorial characteristics	Whole sample (*n* = 400)		Self-reported halitosis				Chi-square value	*P* value
			Yes		No			
	*n*	%	*n*	%	*n*	%		
*Oral hygiene practices*								
Brushing once or less/day	172	43.0	112	57.7	60	29.1	33.36	0.000
Use of chewing stick	51	12.8	29	14.9	22	10.7	1.64	0.201
Changing toothbrush	245	61.2	137	70.6	108	52.4	13.93	0.000
Not using charcoal to clean teeth	365	91.2	171	88.1	194	94.2	4.55	0.033
Not brushing before breakfast	20	5.0	10	5.2	10	4.9	0.02	0.890
Not brushing after breakfast	380	95.0	182	93.8	198	96.1	1.12	0.291
Not brushing before bed	213	53.2	131	67.5	82	39.8	30.84	0.000
Uses toothpaste type TP-B	142	35.5	80	41.2	62	30.1	5.42	0.020
Uses toothpaste type TP-H	101	25.2	32	16.5	69	33.5	15.30	0.000
Uses toothpaste type TP-E	326	81.5	166	85.6	160	77.7	4.13	0.042
Tongue cleaning	166	41.5	110	56.7	56	27.2	35.85	0.000
Use of toothpick	76	19.0	27	13.9	49	23.8	6.32	0.012

*Oral health status*								
Dental hard deposits	128	32.0	93	47.9	35	17.0	43.98	0.000
Food impact between teeth	322	80.5	166	85.6	156	75.7	6.16	0.013
Bleeding gums on tooth brushing	316	79.0	179	92.3	137	66.5	39.97	0.000
Decayed teeth (not yet treated)	184	46.0	104	53.6	80	38.8	8.778	0.003
Loose/mobile teeth	61	15.2	46	23.7	15	7.3	20.87	0.000
Increasing space between teeth	171	42.8	98	50.5	73	34.5	9.28	0.002
Dry mouth	65	16.2	30	15.5	35	17.0	0.17	0.679

*General health problems*								
Hypertension	43	10.8	27	13.9	16	7.8	3.94	0.047
Diabetes	21	5.2	10	5.2	11	5.3	0.01	0.934
Other health/medical problems	87	21.8	47	24.2	40	19.4	1.34	0.244

^#^Each condition presented in this table has basically “Yes and No” alternatives with numerical values corresponding to each individual situation. Only the numerical values corresponding to “Yes” have been presented in this table and the counterpart alternative “No” numerical values have been left out. For example, for the use of chewing stick “Yes versus No,” only the numerical values for “Yes” have been presented in this table while the ones corresponding to “No” have been left out.

**Table 5:** Bivariate analysis: self-reported halitosis in relation to sociodemographic, behavioural, and psychological factors.

^#^Multifactorial characteristics	Whole sample (*n* = 400)		Self-reported halitosis				Chi-square value	*P* value
			Yes		No			
	*n*	%	*n*	%	*n*	%		
*Sociodemographic factors*								
Sex: male	222	55.5	114	58.8	108	52.4	1.62	0.203
Age group: 40 years and above	131	32.8	73	37.6	58	28.2	4.07	0.044
Education level: primary or low	151	37.8	92	47.4	59	28.6	14.99	0.000
Marital status: married	280	70.0	144	74.2	136	66.0	3.21	0.073
Rest place: at home	181	45.2	170	87.6	11	5.3	273.1	0.000
Business place: at work	164	41.0	156	80.4	8	3.9	241.9	0.000
Work environment: outdoor	157	39.2	94	48.5	63	30.6	13.38	0.000

*Behavioural factors *								
Coffee: user	303	75.8	143	73.7	160	77.7	0.85	0.356
Tea: user	380	95.0	183	94.3	197	95.6	0.36	0.551
Alcohol: consumption	188	47.0	97	50.0	91	44.2	1.36	0.243
Cigarette: smoker	83	20.8	50	25.8	33	16.0	5.78	0.016
Chewing gum: user	256	64.0	137	70.6	119	57.8	7.16	0.007
Oral health facility: previous attendance	214	53.5	117	60.3	97	47.1	7.02	0.008

*Psychological factors*								
Met someone with halitosis	45	11.2	9	4.6	36	17.5	16.49	0.000
Do not like the job being done	45	11.2	20	10.3	25	12.1	0.33	0.563
Willing to have a dental check-up	43	10.8	15	7.7	28	13.6	3.58	0.059

^#^Each condition presented in this table has basically “Yes and No” alternatives with numerical values corresponding to each individual situation. Only the numerical values corresponding to “Yes” have been presented in this table and the counterpart alternative “No” numerical values have been left out, for example, sex (male: “Yes,” and female: “No”) and therefore numerical values corresponding to female have been left out.

**Table 6:** Binary logistic regression analyses of the multifactorial characteristics related to self-reported halitosis among the study participants.

Multifactorial characteristics	*B*	S.E.	Odd's ratio	95%	*P* value
				Confidence	
				interval	
*Psychological factors*					
Met someone with bad mouth breath	1.43	0.46	4.19	1.61–9.69	0.003
Hating the type of work one is doing	0.84	0.44	2.31	1.02–5.21	0.044

*Sociodemographic factors*					
Married	−0.61	0.28	0.54	0.31–0.94	0.031
Low level of education	−0.57	0.28	0.56	0.33–0.97	0.040

*Oral health status*					
Have dental problem	−0.95	0.27	0.39	0.23–0.65	0.000
Have dental deposits on the teeth	−1.13	0.29	0.32	0.18–0.57	0.000
Gum bleeding on tooth brushing	−1.59	0.35	0.21	0.10–0.41	0.000
Have loose tooth in the mouth	−0.69	0.41	0.50	0.22–1.11	0.090

*Oral hygiene practices*					
Not brushing teeth before bed	−1.03	0.27	0.36	0.21–0.60	0.000
Not cleaning the tongue	−0.67	0.28	0.51	0.30–0.88	0.016
Not using toothpaste type TP-E	−0.76	0.34	0.47	0.24–0.91	0.025
Not using toothpaste type TP-H	0.53	0.31	1.70	0.93–3.12	0.058
Not brushing after breakfast	1.00	0.58	2.72	0.87–8.50	0.085

*B*: regression coefficient and SE: standard error.

## References

[1] World Health Organization (WHO) (2015). *Health Topics: Oral Health*.

[2] Akaji E. A., Folaranmi N., Ashiwaju O. (2014). Halitosis: a review of the literature on its prevalence, impact and control. *Oral health & preventive dentistry*.

[3] Cortelli J. R., Barbosa M. D. S., Westphal M. A. (2008). Halitosis: a review of associated factors and therapeutic approach. *Brazilian Oral Research*.

[4] Ueno M., Yanagisawa T., Shinada K., Ohara S., Kawaguchi Y. (2007). Prevalence of oral malodor and related factors among adults in Akita Prefecture. *Journal of Medical and Dental Sciences*.

[5] Liu X. N., Shinada K., Chen X. C., Zhang B. X., Yaegaki K., Kawaguchi Y. (2006). Oral malodor-related parameters in the Chinese general population. *Journal of Clinical Periodontology*.

[6] Yaegaki K., Coil J. M. (2000). Examination, classification, and treatment of halitosis; clinical perspectives. *Journal (Canadian Dental Association)*.

[7] Oho T., Yoshida Y., Shimazaki Y., Yamashita Y., Koga T. (2001). Psychological condition of patients complaining of halitosis. *Journal of Dentistry*.

[8] Söder B., Johansson B., Söder P.-Ö. (2000). The relation between foetor ex ore, oral hygiene and periodontal disease. *Swedish Dental Journal*.

[9] Mumghamba E. G., Manji K. P., Michael J. (2006). Oral hygiene practices, periodontal conditions, dentition status and self-reported bad mouth breath among young mothers, Tanzania. *International Journal of Dental Hygiene*.

[10] Morita M., Wang H.-L. (2001). Relationship between sulcular sulfide level and oral malodor in subjects with periodontal disease. *Journal of Periodontology*.

[11] Newman M. G., Takei H., Klokkevold P. R., Carranza F. A. (2011). *Carranza's Clinical Periodontology*.

[12] Bosy A. (1997). Oral malodor: philosophical and practical aspects. *Journal (Canadian Dental Association)*.

[13] Shimura M., Yasuno Y., Iwakura M. (1996). A new monitor with a zinc-oxide thin film semiconductor sensor for the measurement of volatile sulfur compounds in mouth air. *Journal of Periodontology*.

[14] Yaegaki K., Coil J. M. (1999). Clinical application of a questionnaire for diagnosis and treatment of halitosis. *Quintessence International*.

[15] Scully C., Greenman J. (2012). Halitology (breath odour: aetiopathogenesis and management). *Oral Diseases*.

[16] Yaegaki K., Coil J. M., Kamemizu T., Miyazaki H. (2002). Tongue brushing and mouth rinsing as basic treatment measures for halitosis. *International Dental Journal*.

[17] Kida I. A., Manyori C., Masalu J. R. (2010). Prevalence and correlates of perceived oral malodor among adolescents in Temeke district, Dar es Salaam. *East African Journal of Public Health*.

[18] Fadhil O., Mugonzibwa E. (2006). Perception on halitosis among dental patients attending Muhimbili National Hospital dental clinic. *Tanzania Dental Journal*.

[19] Rosenberg M. (1996). Clinical assessment of bad breath: current concepts. *Journal of the American Dental Association*.

[20] ADA Council on Scientific Affairs (2003). Oral malodor. *The Journal of the American Dental Association*.

[21] Sedky N. A. (2015). Perceived impact of halitosis on individual's social life and marital relationship in Qassim Province, KSA. *The Journal of American Science*.

[22] Mumghamba E. G. S., Rutaihwa R. D. (2013). Self-reported, subjectively-determined breath malodor, associated factors, treatment seeking behavior and oral hygiene practices among adults in Kinondoni, Tanzania. *Tanzania Dental Journal*.

[23] Nadanovsky P., Carvalho L. B. M., Ponce De Leon A. (2007). Oral malodour and its association with age and sex in a general population in Brazil. *Oral Diseases*.

[24] Van Den Broek A. M. W. T., Feenstra L., De Baat C. (2008). A review of the current literature on management of halitosis. *Oral Diseases*.

[25] Al-Ansari J. M., Boodai H., Al-Sumait N., Al-Khabbaz A. K., Al-Shammari K. F., Salako N. (2006). Factors associated with self-reported halitosis in Kuwaiti patients. *Journal of Dentistry*.

[26] Paradowska A., Marczewski B., Pawłowska-Cierniak E. (2007). Self-perception of halitosis among students of Wrocław Medical University. *Advances in Clinical and Experimental Medicine*.

[27] Pai V. (2009). Effect of a single-use toothbrush on plaque microflora. *Indian Journal of Dental Research*.

[28] Bello O. O., Osho A., Bankole S. A., Bello T. K. (2013). Antibiotic susceptibility profiles and bacteriological risks associated with used toothbrushes: a case study of some apparently healthy University Students in Southwestern Nigeria. *American International Journal of Biology*.

[29] Peker I., Akarslan Z., Basman A., Haciosmanoglu N. (2015). Knowledge and behavior of dentists in a dental school regarding toothbrush disinfection. *Brazilian Oral Research*.

[30] De Boever E. H., Loesche W. J. (1995). Assessing the contribution of anaerobic microflora of the tongue to oral malodor. *The Journal of the American Dental Association*.

[31] Lindhe J. (1985). Dental plaque and dental calculus. *Textbook of Clinical Periodontology*.

[32] Panagakos F. S., Volpe A. R., Petrone M. E., DeVizio W., Davies R. M., Proskin H. M. (2005). Advanced oral antibacterial/anti-inflammatory technology: a comprehensive review of the clinical benefits of a triclosan/copolymer/fluoride dentifrice. *Journal of Clinical Dentistry*.

[33] Hu D., Zhang Y. P., Petrone M., Volpe A. R., DeVizio W., Giniger M. (2005). Clinical effectiveness of a triclosan/copolymer/sodium fluoride dentifrice in controlling oral malodor: a 3-week clinical trial. *Oral Diseases*.

[34] Sharma N. C., Galustians H. J., Qaqish J. (2007). Clinical effectiveness of a dentifrice containing triclosan and a copolymer for controlling breath odor. *American Journal of Dentistry*.

[35] Eldarrat A., Alkhabuli J., Malik A. (2008). The prevalence of self-reported halitosis and oral hygiene practices among libyan students and office workers. *Libyan Journal of Medicine*.

[36] Tin-Oo M. M., Ying T. Y., Saddki N., Mani S. A. (2013). Self-reported halitosis among medical, dental and health science undergraduate students at the university sains Malaysia. *Acta Stomatologica Croatica*.

[37] Panicker K., Devi R., Honibald E. N., Prasad A. K. (2015). Oral malodor: a review. *Journal of Indian Academy of Dental Specialist Researchers*.

[38] Azodo C., Umoh A. (2013). Self-perceived oral malodour among periodontal patients: prevalence and associated factors. *International Journal of Medicine and Biomedical Research*.

[39] Nalcaci R., Baran I. (2008). Factors associated with self-reported halitosis (SRH) and perceived taste disturbance (PTD) in elderly. *Archives of Gerontology and Geriatrics*.

[40] Youngnak-Piboonratanakit P., Vachirarojpisan T. (2010). Prevalence of self-perceived oral malodor in a group of thai dental patients. *Journal of Dentistry (Tehran)*.

[41] Nalcaci R., Baran I. (2008). Oral malodor and removable complete dentures in the elderly. *Oral Surgery, Oral Medicine, Oral Pathology, Oral Radiology and Endodontology*.

[42] Alshehri F. A. (2016). Knowledge and attitude of Saudi individuals toward self-perceived halitosis. *The Saudi Journal for Dental Research*.

[43] Almas K., Al-Hawish A., Al-Khamis W. (2003). Oral hygiene practices, smoking habit, and self-perceived oral malodor among dental students. *The Journal of Contemporary Dental Practice*.

[44] Settineri S., Mento C., Gugliotta S. C. (2010). Self-reported halitosis and emotional state: impact on oral conditions and treatments. *Health and Quality of Life Outcomes*.

[45] Facco E., Zanette G., Manani G. (2008). Italian version of Corah's Dental Anxiety Scale: normative data in patients undergoing oral surgery and relationship with the ASA physical status classification. *Anesthesia progress*.

[46] Aylıkcı B., Çolak H. (2013). Halitosis: from diagnosis to management. *Journal of Natural Science, Biology and Medicine*.

[47] Morita M., Wang H.-L. (2001). Relationship of sulcular sulfide level to severity of periodontal disease and BANA test. *Journal of Periodontology*.

[48] Aung E. E., Zaitsu T., Ueno M., Kawaguchi Y. (2015). Relationship of oral health knowledge, behavior and status with self-perceived and clinical oral malodor among dental patients. *Journal of Dental Health Oral Disorder & Therapy*.

[49] Dal Rio A. C. C., Nicola E. M. D., Teixeira A. R. F. (2007). Halitosis—an assessment protocol proposal. *Brazilian Journal of Otorhinolaryngology*.

[50] Rosenberg M., Knaan T., Cohen D. (2007). Association among bad breath, body mass index, and alcohol intake. *Journal of Dental Research*.

