# Supplementary material for: Self-Reported Halitosis in relation to Oral Hygiene Practices, Oral Health Status, General Health Problems, and Multifactorial Characteristics among Workers in Ilala and Temeke Municipals, Tanzania
Source: Int J Dent. 2017 Feb 9;2017:8682010. doi: 10.1155/2017/8682010 (PMC5322417; doi:10.1155/2017/8682010)
Supplement: Supplementary file 1 — The file contain the questionnaire used in the study, the questionnaire has open ended and closed ended questions. The questionnaire was formulated in English then translated into Swahili which was the one used for all the participants. [file 8682010.f1.pdf]

QUESTIONNAIRE:

ENGLISH VERSION

RESEARCH TO KNOW ORAL HEALTH STATUS OF WORKERS

PLEASE ANSWER ALL QUESTIONS BY PUTTING THE CORRECT ANSWER IN THE BOX ASIDE.

Serial number [ ][ ][ ]

Q1. Age [ ][ ]

Q2. Sex 1. Male 2. Female [ ]

Q3. Tribe __________________

Q4. Nationality _______________

Q5. Religion _________________

Q6. Date ___________________

Q7. Marital status [ ]
1. Single
2. Married
3. Widow/widower
4. Separated
5. Divorced

Q8. Level of education [ ]
1. No formal education
2. Not completed primary school
3. Primary school
4. Not completed secondary school
5. Secondary education
6. University/college

Q9. What is your occupation?

____

Q10. How much do you like your job? [ ]
1. I like it very much
2. I like it
3. I neither like it nor dislike it
4. I dislike it
5. I dislike it very much

Q11. Are you satisfied with your job? [ ]
1. I am very satisfied
2. I am satisfied
3. I am neither satisfied nor dissatisfied
4. I am dissatisfied
5. I am very dissatisfied.

Q12. Have you ever experienced any dental problem? [ ]
1. Yes
2. No

Q13. Have you ever been to a Dentist?

1. Yes
2. No

Q14. How do you take care of your teeth at home?

1. By brushing
2. No care

Q15. How many times per day do you brush your teeth?

1. Once per day
2. Twice per day
3. Three times per day
4. Four times per day
5. Does not brush at all

Q16. At what time do you brush your teeth?

(a)	In the morning before breakfast	1. Yes	2. No	[ ]
(b)	In the morning after breakfast	1. Yes	2. No	[ ]
(c)	Before lunch	1. Yes	2. No	[ ]
(d)	After lunch	1. Yes	2. No	[ ]
(e)	Before supper	1. Yes	2. No	[ ]
(f)	After supper and before going to bed	1. Yes	2. No	[ ]
(g)	Do not brush	1. Yes	2. No	[ ]

Q17. What type of toothbrush do you use?

(a)	Plastic tooth brush	1. Yes	2. No	[ ]
(b)	Chewing stick	1. Yes	2. No	[ ]

(c) Others, specify__________________

Q18. For how long have you been using the same toothbrush?

1. For 1 month
2. For 2 months
3. For 3 months
4. For 4 months
5. For more than 4 months

Q19. What type of brushing adjuvant do you use?

(a)	Tooth paste	1. Yes	2. No	[ ]
(b)	Charcoal	1. Yes	2. No	[ ]
(c)	Sand	1. Yes	2. No	[ ]
(d)	Ashes	1. Yes	2. No	[ ]

(e) Others, specify____________________

Q20. What type of toothpaste do you use?

(a)	Colgate	1. Yes	2. No	[ ]
(b)	Whitedent	1. Yes	2. No	[ ]
(c)	Aha	1. Yes	2. No	[ ]
(d)	Close up	1. Yes	2. No	[ ]
(e)	Chinese brands	1. Yes	2. No	[ ]
(f)	Chemident	1. Yes	2. No	[ ]
(g)	Sensodyne	1. Yes	2. No	[ ]

(h) Others, specify________________

Q21. Do you have a habit of brushing your tongue?

1. Yes
2. No

Q22. Do you have a problem of getting food impacts between your teeth?

1. Yes
2. No

Q23. If yes to Q 22, what do you use to remove those impacts?

(a) Don't have 1. Yes 2. No [ ]
(b) Toothpick 1. Yes 2. No [ ]
(c) Dental floss 1. Yes 2. No [ ]
(d) Others, specify________________

Q24. In case of no food impacts, do you have the habit of just cleaning the space between the teeth?

1. Yes
2. No

Q25. If yes to Q 24, what do you use?

(a) I don't clean 1. Yes 2. No [ ]
(b) Toothpick 1. Yes 2. No [ ]
(c) Dental floss 1. Yes 2. No [ ]
(d) Others, Specify______________________

Q26. Have you ever met or heard about someone that had a problem of bad breath?

1. Yes
2. No

Q27. Have you ever noted you have bad breath?

1. Yes
2. No

Q28. Does this unpleasant smell bother you?

1. Yes
2. No

Q29. If yes to Q 28, how much does it bother you?

1. It bothers me very much
2. It bothers me
3. It neither bothered nor unbothered
4. I am unbothered
5. I am unbothered very much

Q30. Does your bad breath interfere with your social life?

(a) At home 1. Yes 2. No [ ]
(b) At work 1. Yes 2. No [ ]
(c) I don't have 1. Yes 2. No [ ]
(d) Others, specify

Q31. How did you notice that you have bad breath?

(a) Told by spouse 1. Yes 2. No [ ]
(b) Told by relative 1. Yes 2. No [ ]
(c) Gestures from people near me when talking 1. Yes 2. No [ ]
(d) Don't have 1. Yes 2. No [ ]
(e) Others, specify_______________

Q32. What time of the day you find your breath worst?

(a) In the morning 1. Yes 2. No [ ]
(b) After waking up 1. Yes 2. No [ ]
(c) When talking to other people 1. Yes 2. No [ ]
(d) When fasting 1. Yes 2. No [ ]
(e) During work 1. Yes 2. No [ ]
(f) When thirsty 1. Yes 2. No [ ]
(g) In the afternoon 1. Yes 2. No [ ]
(h) In the evening 1. Yes 2. No [ ]
(i) At night 1. Yes 2. No [ ]
(j) When tired 1. Yes 2. No [ ]
(k) The whole day 1. Yes 2. No [ ]
(l) Don't have 1. Yes 2. No [ ]
(m) Others, specify_______________________

Q33. Would you like your problem to be treated? [ ]

1. Yes
2. No
3. Don't have

Q34. What do you think is the cause of your bad breath?

(a) Dry mouth 1. Yes 2. No [ ]
(b) Bleeding gums 1. Yes 2. No [ ]
(c) Improper brushing of teeth 1. Yes 2. No [ ]
(d) Cavities in teeth 1. Yes 2. No [ ]
(e) Others, Mention ______________________________

Q35. What is /are the causes of bad breath generally?

(a) Improper brushing of the teeth 1. Yes 2. No [ ]
(b) Improper brushing of the tongue 1. Yes 2. No [ ]
(c) Cavities in teeth 1. Yes 2. No [ ]
(d) Bleeding gums 1. Yes 2. No [ ]
(e) Lung diseases 1. Yes 2. No [ ]
(f) Liver dysfunction 1. Yes 2. No [ ]
(g) Dry mouth 1. Yes 2. No [ ]
(h) Others, mention____________________________

Q36. Did you try to manage this problem of bad mouth breath? [ ]

1. Yes
2. No
3. I don't have

Q37. If yes to Q 36, what did you do to manage this problem of bad mouth breath?

(a) I didn't manage 1. Yes 2. No [ ]
(b) I used chewing gum 1. Yes 2. No [ ]
(c) I used Mouth washes 1. Yes 2. No [ ]
(d) I went to see a Doctor 1. Yes 2. No [ ]
(e) Others, specify___________________________

Q38. Do you use chewing gum? [ ]

1. Yes
2. No

Q39. If yes to Q 38, how often?

1. Every day
2. Once per week
3. Twice per week
4. Occasionally
5. Not using

Q40. What is the purpose of using chewing gums?

(a) Freshen the bad mouth breath 1. Yes 2. No [ ]
(b) Enhance salivation 1. Yes 2. No [ ]
(c) Just for pleasure 1. Yes 2. No [ ]
(d) Not using 1. Yes 2. No [ ]
(e) Others, specify____________

Q41. Do you have any of the following problems as a result of your bad breath?

(a) I hesitate to talk to people 1. Yes 2. No [ ]
(b) Do not like to meet other people 1. Yes 2. No [ ]
(c) Other people avoid me 1. Yes 2. No [ ]
(d) None 1. Yes 2. No [ ]
(e) Others, specify____________________

Q42. Have you ever experienced or experiencing bleeding gums on brushing?

1. Yes
2. No

Q43. If yes to Q 42, what actions did you take?

(a) I did nothing but continue brushing 1. Yes 2. No [ ]
(b) I stopped brushing 1. Yes 2. No [ ]
(c) I went to a Dentist 1. Yes 2. No [ ]
(d) I went to a physician 1. Yes 2. No [ ]
(e) I went to a traditional healer 1. Yes 2. No [ ]
(f) Others, specify____________________

Q44. Do you have any Health problem?

1. Yes
2. No

Q45. If yesto Q 49, what are they?

(a) Diabetes 1. Yes 2. No [ ]
(b) Hypertension 1. Yes 2. No [ ]
(c) Tuberculosis 1. Yes 2. No [ ]
(d) Others, specify ______________________

Q46. Have you ever-experienced dry mouth?

1. Yes
2. No

Q47. Do you smoke?

1. Yes
2. No

Q48. At what time do you normally smoke?

1. Every day
2. Very often
3. Whole of the time
4. Not smoking
5. Others, specify____________________

Q49. How many cigarettes do you smoke per day?

1. 1-5 pieces
2. 6-10 pieces
3. 11-15 pieces
4. 16-20 pieces
5. 20+ pieces
6. Not smoking

Q50. Do you drink alcohol?

1. Yes
2. No

Q51. How frequently do you drink alcohol?

1. Once per week
2. Every day
3. Occasionally
4. Not drinking
5. Others, specify__________________

Q52. Do you normally drink Tea?

1. Yes
2. No

Q53. Do you drink coffee?

1. Yes
2. No

Q54. Do you have any tooth that is mobile at present?

1. Yes
2. No

Q55. Have you noticed any hard deposits on your teeth?

1. Yes
2. No

Q56. Do you have any extracted tooth?

1. Yes
2. No

Q57. How many teeth were extracted due to holes/cavities only?

Q58. How many teeth were extracted due to mobility only?

Q59. How many teeth were extracted due to Trauma/Accidents only?

Q60. Have you ever noticed any change of alignment e.g. developing spaces between teeth which were not there before?

1. Yes
2. No

THANK YOU FOR YOUR COOPERATION

DODOSO

UTAFITI KUJUA JINSI WAFANYAKAZI WANAVYOTUNZA AFYA YA KINYWA NA MENO

TAFADHALI JIBU MASWALI YOTE KWA KUWEKA JIBU SAHIHI KWENYE KISANDUKU PEMBENI MWA SWALI

Namba ya dodoso

S1. Umri

S2. Jinsia 1. Kiume 2. Kike

S3. Kabila ___________________

S4. Utaifa __________________

S5. Dini __________________

S6. Tarehe __________________

S7. Hali ya ndoa

1. Sijaolewa/sijaoa
2. nimeolewa/nimeoa
3. mjane
4. Tumetengana
5. Tumetalikiana

S8. Kiwango chako cha elimu

1. sijasoma kabisa
2. Sikumaliza elimu ya msingi
3. Elimu ya msingi
4. Sikumaliza elimu ya sekondari
5. elimu ya sekondari
6. elimu ya chuoni

S9. Je unafanya kazi gani?

____

S10. Je unaipenda kazi yako?

1. Naipenda sana
2. Naipenda
3. Siipendi wala siichukii
4. siipendi
5. Siipendi kabisa

S11. Je unaridhika na kazi unayoifanya?

1. Naridhika sana
2. Naridhika
3. Siridhiki wala siichukii
4. Siridhiki
5. Sirikiki kabisa

S12. Ulishawahi kupata tatizo lolote la meno?

1. Ndiyo
2. Hapana

S13. Umeshawahi kwenda kwa Daktari wa Meno?

1. Ndiyo
2. Hapana

S14. Unayatunzaje meno yako nyumbani?

1. Kwa kupiga mswaki
2. Sipigi mswaki

S15. Unapiga mswaki mara ngapi kwa siku?

1. Mara moja kwa siku
2. Mara mbili kwa siku
3. Mara tatu kwa siku
4. Mara nne kwa siku
5. sipigi mswaki kabisa

S16. Ni wakati gani unapiga mswaki?

(a) Asubuhi kabla ya kifungua kinywa	1. Ndiyo 2. Hapana	[ ]
(b) Asubuhi baada ya kifungua kinywa	1. Ndiyo 2. Hapana	[ ]
(c) Kabla ya chakula cha mchana	1. Ndiyo 2. Hapana	[ ]
(d) Baada ya chakula cha mchana	1. Ndiyo 2. Hapana	[ ]
(e) kabla ya chakula cha usiku	1. Ndiyo 2. Hapana	[ ]
(f) Baada ya chakula cha usiku, kabla ya kulala	1. Ndiyo 2. Hapana	[ ]
(g) Sipigi mswaki kabisa	1. Ndiyo 2. Hapana	[ ]

S17. Ni aina gani ya mswaki unaoutumia?

(a) Mswaki wa plastiki	1. Ndiyo 2. Hapana	[ ]
(b) Mswaki wa mti	1. Ndiyo 2. Hapana	[ ]

(c) Mingineyo, taja____________________

S18. Unatumia mswaki mmoja bila kubadili kwa muda gani?

1. Kwa mwezi mmoja
2. Kwa miezi miwili
3. Kwa miezi mitatu
4. Kwa miezi mine
5. Kwa miezi zaidi ya minne

S19. Unatumia nini kusafisha meno yako?

(a) Dawa ya meno	1. Ndiyo 2. Hapana	[ ]
(b) Mkaa	1. Ndiyo 2. Hapana	[ ]
(c) Mchanga	1. Ndiyo 2. Hapana	[ ]
(d) Majivu	1. Ndiyo 2. Hapana	[ ]

(e) Nyinginezo, taja_____________________

S20. Unatumia dawa ya meno ya aina gani?

(a) Kolgate	1. Ndiyo	2. Hapana	[ ]
(b) Waitidenti	1. Ndiyo	2. Hapana	[ ]
(c) Aha	1. Ndiyo	2. Hapana	[ ]
(d) Klozi apu	1. Ndiyo	2. Hapana	[ ]
(e) Dawa za Kichina	1.Ndiyo	2.Hapana	[ ]
(f) Kemidenti	1.Ndiyo	2. Hapana	[ ]
(g) Sensodaini	1. Ndiyo	2. Hapana	[ ]

(h) Nyinginezo, taja___________________________________

S21.Je una kawaida ya kuswaki ulimi wako?

1. Ndiyo
2. Hapana

S22. Una tatizo la chakula kubaki/kuganda katikati ya meno yako?

1. Ndiyo
2. Hapana

S23. Kama ndiyo kwa S 22, Unatumia nini kutoa hayo mabaki?

(a) Sina mabaki 1. Ndiyo 2. Hapana [ ]
(b) Vijiti vya meno 1. Ndiyo 2. Hapana [ ]
(c) Uzi au kamba maalumu 1. Ndiyo 2. Hapana [ ]
(d) Nyinginezo, taja_____________________________________

S24. kama hakuna mabaki katikati ya meno, unakawaida ya kusafisha nafasi katikati ya meno yako?

1. Ndiyo
2. Hapana

S25. Kama Ndiyo kwa S 24, unatumia nini?

(a) Sisafishi 1. Ndiyo 2. Hapana [ ]
(b) Vijiti vya meno 1. Ndiyo 2. Hapana [ ]
(c) Uzi au kamba maalumu 1. Ndiyo 2. Hapana [ ]
(d) Nyinginezo,taja___________________________________

S26. Umeishawahi kukutana au kusikia mtu anatatizo la harufu isiyopendeza kutoka kinywani?

1. Ndiyo
2. Hapana

S27. Umewahi kupata tatizo la harufu isiyopendeza kutoka kinywani mwako?

1. Ndiyo
2. Hapana

S28. Je unakerwa na harufu hiyo isiyopendeza kutoka kinywani mwako?

1. Ndiyo
2. Hapana

S29. Kama ndiyo kwa S 28, unakerwa kiasi gani na harufu isiyopendeza kutoka kinywani mwako?

1. Nakerwa sana
2. Nakerwa
3. Sikerwi /nakerwa
4. Sikerwi
5. Sikerwi kabisa

S30. Je harufu hiyo isiyopendeza kutoka kinywani mwako inaathiri maisha yako ya kila siku ?

(a) Nyumbani 1. Ndiyo 2. Hapana [ ]
(b) Kazini 1. Ndiyo 2. Hapana [ ]
(c) Sina harufu 1. Ndiyo 2. Hapana [ ]
(d) Kwingineko. Taja_____________________________

S31. Je ulifahamu vipi kuwa una harufu isiyopendeza kutoka kinywani mwako?

(a) Niliambiwa na mwenzangu 1. Ndiyo 2. Hapana [ ]
(b) Niliambiwa na ndugu 1. Ndiyo 2. Hapana [ ]
(c) Matendo ya watu walio karibu nami ninapozungumza 1. Ndiyo 2. Hapana [ ]
(d) Sina harufu 1. Ndiyo 2. Hapana [ ]
(e) Nyinginezo, taja_____________________________________

S32. Je ni wakati gani hasa unapata harufu isiyopendeza kutoka kinywani?

(a) Wakati wa asubuhi 1. Ndiyo 2. Hapana [ ]
(b) Mara baada ya kuamka 1. Ndiyo 2. Hapana [ ]
(c) Ninapoongea na wengine 1. Ndiyo 2. Hapana [ ]
(d) Nikiwa nimefunga 1. Ndiyo 2. Hapana [ ]
(e) Wakati wa kazi 1. Ndiyo 2. Hapana [ ]
(f) Ninapokuwa na kiu 1. Ndiyo 2. Hapana [ ]
(g) wakati wa mchana 1. Ndiyo 2. Hapana [ ]
(h) Wakati wa jioni 1. Ndiyo 2. Hapana [ ]
(i) Usiku 1. Ndiyo 2. Hapana [ ]
(j) Ninapokuwa nimechoka 1. Ndiyo 2. Hapana [ ]
(k) Wakati wote 1. Ndiyo 2. Hapana [ ]
(l) Sitoi harufu 1. Ndiyo 2. Hapana [ ]
(m) Nyinginezo, taja ___________________________________

S33. Je unapenda kutibiwa tatizo lako?

1. Ndiyo
2. Hapana
3. sina harufu

[ ]

S34. Unafikiri nini chanzo cha harufu isiyopendeza kutoka kinywani mwako?

(a) Kukaukwa na mate 1. Ndiyo 2. Hapana [ ]
(b) Fizi zitoazo damu 1. Ndiyo 2. Hapana [ ]
(c) Kutopiga mswaki vizuri 1. Ndiyo 2. Hapana [ ]
(d) Meno yaliyotoboka 1. Ndiyo 2. Hapana [ ]
(e) Nyinginezo, taja _________________________________

S35. Je nini chanzo/vyanzo cha/vya harufu isiyopendeza kutoka kinywani kwa ujumla?

(a) Kutopiga mswaki vizuri 1. Ndiyo 2. Hapana [ ]
(b) Kutokuswaki ulimi vizuri 1. Ndiyo 2. Hapana [ ]
(c) Meno yaliyotoboka 1. Ndiyo 2. Hapana [ ]
(d) Fizi zitoazo damu 1. Ndiyo 2. Hapana [ ]
(e) Magonjwa ya mapafu 1. Ndiyo 2. Hapana [ ]
(f) Matatizo ya ini 1. Ndiyo 2. Hapana [ ]
(g) Kukaukwa na mate 1. Ndiyo 2. Hapana [ ]
(h) Nyinginezo, taja_______________________________

S36. Je ulishawahi kujaribu kujitibia tatizo la harufu isiyopendeza kutoka kinywani?

1. Ndiyo
2. Hapana
3. Sina harufu

[ ]

S37. Kama Ndiyo kwa S 36, ulijitibiaje?

(a) Sikujitibu 1. Ndiyo 2. Hapana [ ]
(b) Nilitumia bazooka 1.Ndiyo 2. Hapana [ ]
(c) Nilitumia dawa za kusukutua 1. Ndiyo 2. Hapana [ ]
(d) Nilienda kwa Daktari 1. Ndiyo 2. Hapana [ ]
(e) Nyinginezo, taja ______________________________

S38. Unatumia bazoka?

1. Ndiyo
2. Hapana

[ ]

S39. Kama Ndiyo kwa S 38, mara ngapi?

1. Kila siku
2. Mara moja kwa wiki
3. Mara mbili kwa wiki
4. Kwa matukio maalumu
5. Situmii kabisa

S40. Nini madhumuni ya kutumia bazoka?

(a) Kuondoa harufu isiyopendeza inayotoka kinywani 1. Ndiyo 2. Hapana [ ]
(b) Kusababisha mate yaongezeke 1. Ndiyo 2. Hapana [ ]
(c) Kwa kujifurahisha 1. Ndiyo 2. hapana [ ]
(d) situmii kabisa 1. Ndiyo 2. Hapana [ ]
(e) nyinginezo, taja_______________________________________

S41. Je una tatizo kati ya haya yafuatayo yanayosababishwa na harufu isiyopendeza kutoka kinywani?

(a) Kusita kuongea na watu 1. Ndiyo 2. Hapana [ ]
(b) Kutopenda kukutana na watu 1. Ndiyo 2. Hapana [ ]
(c) Watu wengine wananiepuka 1. Ndiyo 2. Hapana [ ]
(d) sina tatizo 1. Ndiyo 2. Hapana [ ]
(e) mengineyo,taja________________

S42. Umeshawahi kutokwa na damu kwenye fizi wakati wa kupiga mswaki?

1. Ndiyo
2. Hapana

S43. Kama Ndiyo kwa S 42, ulichukua hatua gani?

(a) Sikufanya chochote ila niliendelea kupiga mswaki 1. Ndiyo 2. Hapana [ ]
(b) Niliacha kupiga mswaki 1. Ndiyo 2. Hapana [ ]
(c) nilienda kwa daktari wa meno 1. Ndiyo 2. Hapana [ ]
(d) nilienda kwa daktari wa kawaida 1. Ndiyo 2. Hapana [ ]
(e) nilienda kwa mganga wa kienyeji 1. Ndiyo 2. Hapana [ ]
(f) nyinginezo, taja__________________________

S44. Je una tatizo jingine lolote la kiafya?

1. Ndiyo
2. Hapana

S45. Kama Ndiyo kwa S 44, ni lipi?

(a) Kisukari 1. Ndiyo 2. Hapana [ ]
(b) Shinikizo la damu 1. Ndiyo 2. Hapana [ ]
(c) Kifua kikuu 1. Ndiyo 2. Hapana [ ]
(d) Mengineyo, taja__________________

S46. Ulishawahi kupata na hali ya kukaukwa na mate?

1. Ndiyo
2. Hapana

S47. Unavuta sigara?

1. Ndiyo
2. Hapana

S48. Unavuta sigara wakati gani?
1. Kila siku
2. Mara moja moja
3. Wakati wote
4. Sivuti
5. Mengineyo, taja______________________

S49. Unavuta sigara ngapi au mitemba/misokoto mingapi kwa siku?
1. Sivuti
2. 1-5
3. 6-10
4. 11-15
5. 16-20
6. zaidi ya 20

S50. Unakunywa pombe?
1. Ndiyo
2. Hapana

S51. Unakunywa pombe mara ngapi?
1. Mara moja kwa wiki
2. Kila siku
3. Kwa wakati maalumu
4. Sinywi
5. mengineyo, taja__________________

S52. Una kawaida ya kunywa chai?
1. Ndiyo
2. Hapana

S53. Unakunywa kahawa?
1. Ndiyo
2. Hapana

S54. Unalo jino linalolegea kwa sasa?
1. Ndiyo
2. Hapana

S55. Umewahi kuona/kuhisi kama una magamba magumu(ugaga) kwenye meno yako?
1. Ndiyo
2. Hapana

S56. Ulishawahi kung'oa jino/meno kabla?
1. Ndiyo
2. Hapana

S57. Ni meno mangapi yaling'olewa sababu ya kutoboka tu?

S58. Ni meno mangapi yaling'olewa sababu ya kulegea tu?

S59. Ni meno mangapi yaling'olewa sababu ya kuumia/ajali tu?

S60. Ulishawahi kuona mabadiliko ya mpangilio wa meno yako mfano kuwa na nafasi kwenye meno ambazo hazikuwepo kabla?

1. Ndiyo
2. Hapana

NASHUKURU SANA KWA USHIRIKIANO WAKO
